# Nutrient estimation from an FFQ developed for a black Zimbabwean population

**DOI:** 10.1186/1475-2891-4-37

**Published:** 2005-12-13

**Authors:** Anwar T Merchant, Mahshid Dehghan, Jephat Chifamba, Getrude Terera, Salim Yusuf

**Affiliations:** 1 Population health Research Institute, McMaster University, Hamilton ON, Canada; 2 Department of Clinical Epidemiology and Biostatistics, McMaster University, Hamilton ON, Canada; 3 Department of Medicine, McMaster University, Hamilton ON, Canada; 4 Department of Physiology, University of Zimbabwe College of Health Sciences, Harare, Zimbabwe

## Abstract

**Background:**

There is little information in the literature on methods of food composition database development to calculate nutrient intake from food frequency questionnaire (FFQ) data. The aim of this study is to describe the development of an FFQ and a food composition table to calculate nutrient intake in a Black Zimbabwean population.

**Methods:**

Trained interviewers collected 24-hour dietary recalls (24 hr DR) from high and low income families in urban and rural Zimbabwe. Based on these data and input from local experts we developed an FFQ, containing a list of frequently consumed foods, standard portion sizes, and categories of consumption frequency. We created a food composition table of the foods found in the FFQ so that we could compute nutrient intake. We used the USDA nutrient database as the main resource because it is relatively complete, updated, and easily accessible. To choose the food item in the USDA nutrient database that most closely matched the nutrient content of the local food we referred to a local food composition table.

**Results:**

Almost all the participants ate sadza (maize porridge) at least 5 times a week, and about half had matemba (fish) and caterpillar more than once a month. Nutrient estimates obtained from the FFQ data by using the USDA and Zimbabwean food composition tables were similar for total energy intake intra class correlation (ICC) = 0.99, and carbohydrate (ICC = 0.99), but different for vitamin A (ICC = 0.53), and total folate (ICC = 0.68).

**Conclusion:**

We have described a standardized process of FFQ and food composition database development for a Black Zimbabwean population.

## Introduction

Diet is central in the development of obesity and chronic diseases and is changing rapidly in low and middle income countries [1]. Some of the constraints in studying diet and its correlates in poor countries is that diet varies considerably from place to place and among different socio-economic classes, its assessment methods have evolved relatively recently (1980s and 1990s), and have not been developed for those populations [2]. Moreover, the technical expertise and financial resources to develop these instruments may be lacking. We are conducting a longitudinal investigation in about 120000 people in 14 countries at different stages of development, one of which is Zimbabwe, measuring diet with an FFQ. The FFQ is commonly used for nutritional assessment in large epidemiologic studies, because it measures long-term diet, is quick and comparatively inexpensive to administer, and provides quantitative information on nutrients and foods [2]. To calculate nutrient intake from an FFQ we need to have data from a food composition database that lists the nutrient content of the foods contained in the FFQ. This is critical because the nutrient content of the same food can vary substantially [3]. However, the food composition database that we found for Zimbabwe was 24 years old and was not updated regularly [4]. Moreover, many nutrients were not estimated at all. Therefore we could not use the Zimbabwean food composition database to estimate nutrient intake from the FFQ. We thus created a food composition database for Zimbabwe using the Zimbabwe and USDA nutrient databases. In this paper we briefly describe the development of a semi-quantitative FFQ, and creation of food composition table from USDA database for Black Zimbabwean population.

## Subjects and methods

### Study population

The urban participants lived in Harare, either in a relatively affluent area, or in an urban low-income area; the rural participants lived in Chidamoyo, 345 km from Harare. All participants were = 35 years of age, and free of any reported co-morbid conditions. The study was approved by the Institutional Review Boards of the participating institutions and the relevant authorities.

### Development of FFQ

There were three major steps in FFQ development. First, we prepared a list of commonly eaten foods in Zimbabwe, including estimates of the usual portion sizes and frequencies of intake. Second, based on this list we developed a long FFQ. Third, we tested the long FFQ in the field and shortened it. We are using this version of the FFQ in our study and are validating by comparing it with multiple 24-hour dietary recalls.

#### Development of the food list

We used three approaches to create a food list: we conducted a 24-hour dietary recall in November 2003 among 200 persons in urban and rural areas. Second, we added foods to this list that were commonly eaten in that Zimbabwean population at other times of the year and on special occasions. Third, we further expanded this list by adding foods that were nutrient rich but were not captured in the previous steps (caterpillar for example) from a previously prepared food composition table for Zimbabwe and the observations of the local Zimbabwean nutritionists.

We then formatted the foods on the expanded list into questions. We organized the foods items based on their similarities in nutrient content into 6 major food groups as has been done elsewhere [5]. The food groups were: 1. Bread, cereal and starches (different types of bread, sadza, rice, pasta, porridge and bakery products), 2. Meat and eggs(including insects and sea food), 3. Dairy products, 4. Vegetables (fresh and cooked vegetables excluding potatoes), and fruits (fresh and canned), 5. Beverages (fruit juice, alcoholic and non-alcoholic drinks, coffee and tea), 6. Sweets and baked goods (nuts, candy and cake).

#### Frequency of intake

To estimate average frequency of intake of most foods in the previous year we used closed-ended responses consisting of 9 categories: Never or less than once/month, 1–3/m, 1/week, 2–4/wk, 5–6/wk, 1/day, 2–3/d, 4–5/d, >6/d [5].

#### Portion size

To obtain food portion size, we physically examined all reported portions and took the most commonly consumed portion as the unit of measurement.

#### Seasonality

Unlike in most western countries fruit and vegetable availability and cost vary greatly by season in Zimbabwe. We asked how often on average fruits and vegetables with seasonal variation were consumed in season. Our colleagues in Zimbabwe prepared the list of seasonal fruits and vegetables and estimated the length of season for each item. This allowed us to take seasonality into account to calculate the average daily intakes of such foods.

#### Testing of pilot FFQ

We pilot tested the long FFQ by administering it to 100 participants in July 2004, and excluded infrequently eaten foods.

### Food composition database

The food composition database that we found for Zimbabwe was 24 years old and was not updated regularly. Moreover, the reported nutrients in the food composition table included those recommended by Southgate in 1974 [4]. Therefore we could not use the Zimbabwean food composition database to estimate nutrient intake from the FFQ. We therefore created a food composition database for Zimbabwe using the Zimbabwe and USDA nutrient databases.

However, the USDA nutrient database has many different entries for the same food with different nutrient compositions. If we arbitratily chose any one of those foods its nutrient composition might have been different from that of the same food used in Zimbabwe. We therefore used the Zimbabwen food composition database to help us choose the same food item from the USDA nutrient databse that was most similar in nutrient composition to the one used in Zimbabwe. For example, among 13 apples in USDA nutrient database, Apples, raw, with skin code 9003 was the most similar to the apple in Zimbabwe food composition table (Table 1). We compared the energy, macronutrient and some micronutrient content of each food item reported in both food composition tables and chose the food from the USDA nutrient database that was closest in nutrient content to the food described in the Zimbabwean food composition table (Table 2).

**Table 1:** Comparison of nutrient composition of an apple described in the local Zimbabwe food composition table with different apples in the USDA nutrient database

NDB No	Shrt_Desc	Water/g	Energ/Kcal	Protein/g	Lipid/g	CHO/g	Ca/mg	Fe/mg	P/mg	K/mg	Na/mg	Vit_C/mg	Vit B1/mg	Vit B2/mg	Niacin/mg	Vit B6/mg	Folate/mg	Vit_A RAE
09003	Apple, raw w Skin	85.56	52	0.26	0.17	13.81	6	0.12	11	107	1	4.6	0.017	0.026	0.091	0.041	3	3
09004	Apple raw wo, skin	86.67	48	0.27	0.13	12.76	5	0.07	11	90	0	4	0.019	0.028	0.091	0.037	0	2
09005	Apple raw wo, skin Ckd bld	85.47	53	0.26	0.36	13.64	5	0.19	8	88	1	0.2	0.016	0.012	0.095	0.044	1	2
09006	Apple raw wo, skin / ckd microwave	84.63	56	0.28	0.42	14.41	5	0.17	8	93	1	0.3	0.017	0.011	0.061	0.046	1	2
09007	Apple cnd, swtnd, sliced, dr nd, unhtd	82.36	67	0.18	0.49	16.7	4	0.23	5	68	3	0.4	0.009	0.01	0.073	0.044	0	3
09008	Apple cnd, swtnd Sliced, drnd, htd	82.28	67	0.18	0.43	16.84	4	0.24	6	70	3	0.2	0.009	0.01	0.081	0.044	0	3
09009	Apple, Dehyd (lo moist) sulfured unckd	3	346	1.32	0.58	93.53	19	2	55	640	124	2.2	0.046	0.13	0.68	0.28	1	4
09010	Apple, Dehyd (lo moist) sulfured stwd	79.36	74	0.28	0.12	19.91	4	0.43	12	136	26	0.6	0.008	0.029	0.14	0.054	0	1
09011	Apple, dried sulfured, unckd	31.76	243	0.93	0.32	65.89	14	1.4	38	450	87	3.9	0	0.159	0.927	0.125	0	0
09012	Apple, dried sulfured, stwd, wo/sugar	84.13	57	0.22	0.07	15.32	3	0.33	9	105	20	1	0.006	0.019	0.129	0.05	0	1
09013	Apple, dried sulfured, stwd, w/sugar	78.76	83	0.2	0.07	20.73	3	0.31	8	98	19	0.9	0.006	0.018	0.121	0.047	0	1
09014	Apple, frz, unswtnd, unhtd	86.85	48	0.28	0.32	12.31	4	0.18	8	77	3	0.1	0.013	0.011	0.042	0.034	1	2
09015	Apple, frz, unswtnd, htd	87.16	47	0.29	0.33	12	5	0.19	8	76	3	0.4	0.014	0.011	0.043	0.032	1	1
	Apple_Zimbabwe	84.6	56.2	0.4	0.6	13.1	6.5	0.6	10.8	106	1.5	5.4	0.03	0.03	0.15	0.03	3.9	13.39

**Table 2:** Comparison of nutrient content of some foods calculated from Zimbabwe and USDA food composition tables

**Foods**	**Code**	**Energy kcal**	**Protein g**	**Fat g**	**CHO g**	**Ca mg**	**P mg**	**Fe mg**	**K mg**	**Na mg**	**Vit† A RE**	**Vit C mg**	**B1 mg**	**B2 mg**	**B3 mg**	**B6 mg**	**B12 mg**	**Folate mg**
**Corn flour**	Zim_FCT*	356.2	4.5	2.1	84.0	7.0	45.7	1.6	30.5	26.0	0	0	0.01	0	0.10	tr**	0	tr
Corn, white	20314	365	9.42	4.74	74.2	7	210	2.71	287	35	0	0			3.627	0.622	0	
**Corn flour, whole-grain, white**	**20316**	**361**	**6.93**	**3.86**	**76.85**	**7**	**272**	**2.38**	**315**	**5**	**0**	**0**			**1.900**	**0.370**	**0**	**25**
Cornmeal, whole-grain, white	20320	362	8.12	3.59	76.89	6	241	3.45	287	35	0	0			3.632	0.304	0	25
Shakata (Mobola Plum) this must be a mistake		167.5	1.2 0	0.5	41.9			2.2			158.3	55.7		0.5				
Plums, raw	09279	46	0.70	0.28	11.42	6	16	0.17	157	0	17	9.5			0.417	0.029	0	5
Plums, canned, purple, water pack, solids and liquids	09281	41	0.39	0.01	11.03	7	13	0.16	126	1	46	2.7			0.370	0.027	0	3
Plums, canned, purple, light syrup pack, solids and liquids	09283	63	0.37	0.10	16.28	9	13	0.86	93	20	12	0.4			0.297	0.027	0	3
Guavas this also		66	1	0.5	14.6	16.6	26.0	0.9	290	4.0	50.0	221.4	0.05	0.04	1.1		0.0	
Guavas, common, raw	09139	68	2.55	0.95	14.32	18	40	0.26	417	2	31	228.3			1.084	0.110	0	49
Chicken raw	Zim_FCT	195.1	19	12.9	0	12	179	1.3	227	68.3	105	0.9	0.8	0.15	7.7	0.3	0.4	5
Chicken raw	5006	**215**	**18.6**	**15.1**	0	**11**	147	0.9	189	**70**	41	**1.6**	0.06	0.12	**6.8**	0.35	**0.3**	**6**
Chicken raw, meat and skin stewing	5123	258	17.6	20.3	1.04	10	**172**	**1.04**	**204**	**71**	52	0	**0.11**	**0.17**	6.26	0.33	**0.3**	**6**
Chicken roast, skin and meat	Zim_FCT	216	26.7	8.2	0	12	214	1.5	308	82.4	45	0	0.7	0.23	5.6			
Chicken roast, skin and meat	5111	216	17.1	15.9	0	10	166	1.01	196	68	38	0	0.06	0.12	6.57	0.32	0.3	6
Chicken roasted, skin and meat	5055	239	28.2	13.2	0	24	165	1.39	237	96	28	0	0.07	0.22	7.069	0.34	0.3	7
Chicken, cooked, roasted	5069	**216**	**27**	11.2	0	**12**	**175**	**1.33**	**229**	**90**	30	0	0.07	**0.22**	**5.994**	0.34	0.3	8
Spinach (Mova)	Zim_FCT	15.9	1.2	0.2	2.4	33.1	30.3	0.9	228.3	8.9	419.4	11.10	0.07	0.08	0.35	0.06	0	32.80
Malabar, Spinach, cooked	11986	23	2.98	0.78	2.71	124	36	1.48	256	55	58	5.9			0.787	0.086	0	114
New Zealand spinach, cooked, boiled, drained, without salt	11277	12	1.30	0.17	2.20	48	22	0.66	102	107	181	16.0			0.390	0.237	0	8

* Zim_FCT = Zimbabwe food composition table

** Tr = trace

† Vit = vitamin

For mixed dishes we entered local recipes into an Excel spreadsheet and applied yield and retention factors taken form USDA Handbook No.102 (food yields, summarized by different stages of preparation) [3] to obtain the nutrient composition for given portions of Zimbabwean mixed dishes, thus accounting for the method of food preparation. We then estimated nutrient content for the most commonly used portion of that mixed dish.

For those food items, which were not available in the USDA nutrient database such as caterpillar, we chose a food item from the same food category (sushi caterpillar) [6] and imputed those nutrient values in our database for caterpillar.

Finally, for some other foods which were not available in USDA we found the common name, scientific name of local food items and matched them with the same scientific name in English and then chose similar food item from USDA. For example, taro is *Colacasia antiquorum *and is from the yam family. Therefore, we imputed yam nutrient content for taro.

#### Recipe analysis

To calculate the nutrient content of mixed dish gathered local recipes as described elsewhere [7]. There were 112 recipes in the recipe database and we calculated the nutrient content of those recipes based on the chosen food items from USDA, as described in the previous section. We did not choose enriched foods items to estimate nutrient content because these are not generally used in Zimbabwe. In nutrient estimation we took into account the loss of minerals, vitamins and moisture in food preparation, such as boiling, frying or steaming. From this information we were able to calculate the nutrient content of 100 g or a portion of food.

### Data management and statistical analysis

We entered information from the FFQ and basic demographic characteristics of the participants into SPSS. We calculated the frequency of intake of a portion of each food on the FFQ. We administered the FFQ to 100 people and estimated the mean and standard deviation (SD) of nutrient intake from 30 commonly eaten foods using the USDA and Zimbabwean food composition tables. To compare the estimates we calculated intraclass correlations.

## Results

Of the 200 persons who responded to the 24 hr Dr and FFQ slightly more than half were women (Table 3). The mean age was nearly 51 years and ranged from 34–93 years. Many people (40%) did not respond to the question on income. Among the respondents more than half reported incomes in the very low category. There were few illiterate or never married persons in this sample. The mean age for those who did not report their income was 54 ± 15.4 years old and they were mostly illiterate or had only primary school education (16.1% illiterate, 54% primary and 30% secondary level education).

**Table 3:** Characteristics of subjects who participated in 24 hr dietary recall and pilot testing Long-FFQ*

	**FFQ**	**24 hr DR**
**Women**	N = 55	N = 43
Age (years)	50.6 (12.1)	52.3 (14.4)
BMI (kg/m^2^)	26.3 (4.8)	23.6 (5.0)
**Men**	(N = 43)	N = 45
Age (years)	51.4 (16.1)	51 (13.6)
BMI (kg/m^2^)	23.7 (6.0)	21.8 (3.6)
Income (ZW $)**		
Very-Low	22 (52.4%)	63 (88.7%)
Low	15 (35.7%)	7 (9.9%)
Middle	5 (11.9%)	1 (1.4%)
No response	56	24
**Marital status**		
Never married	3 (3%)	4 (4.5%)
Married	72 (72.2%)	48 (53.9%)
Widow, divorced or separated	25 (24.2%)	37 (41.6%)
**Education**		
Illiterate	10 (10.1%)	20 (22.5%)
Primary or secondary school	87 (87.8%)	61 (68.6%)
University, college	2 (2%)	5 (5.6%)
**Profession**		
Professionals	1 (1.3%)	4 (5.6%)
Technicians and associated worker	17 (22.1%)	3 (4.2%)
Elementary occupation	22 (28.6%)	40 (56.3%)
Homemaker	37 (48.1%)	3 (4.2%)

* The numbers in each of the categories in this table are different because the number of reponses we received varied

** Exchange rate US $ 1 = 5300 Zimbabwe dollar

Very low < 3 × 10^5 ^ZW $/month

Low 3 × 10^5 ^to 1 × 10^6 ^ZW $/month

Middle 1 × 10^6^to 2.5 × 10^6 ^ZW $/month

High >2.5 × 10^6 ^ZW $/month

The staple food in this population was maize. It was cooked in different ways such as sadza, samp, or mealie porridge (Table 4). Lacto, a commercially produced fermented milk product, was a frequently consumed dairy food. Certain types of caterpillars were eaten, either dried or fried. Matemba (small fish) were preserved and eaten in small quantities with other food like condiments. In addition people ate many fruits and vegetables unique in that area (Table 4). A copy of the FFQ is in Appendix 1.

**Table 4:** Description of some foods unique to Zimbabwe

**Food**	**Description**
	
**Bread, cereal and starches**	
Sadza	Stiff porridge (prepared from meal of maize, millet, sorghum or rice) and contain a small amount of fibre. The meal of maize is the staple food in Zimbabwe
Samp (Mashakada*)	Boiled maize grain (previously dried), sometimes its degerminated broken grain, pounded and boiled
Mealie Porridge	Ground corn, boiled not too thick, if more is added becomes sadza, other foods can be added like peanut butter (sometimes margarine), sugar (amounts depends on quantity), milk, some people even add bread
Pumpkin Porridge African bread	Mash prepared from cattle melon or pumpkin ingredients: corn flour, egg, sugar and water
	
**Fruits**	
Baobab	Adansonia digitata
Paw Paw	Carica Papaya, Papaya
Naartjie	Citrus aurantium, Tangerine
Shakata	Parinari Curatellifolia, Mobola Plum
	
**Vegetables**	
Gourd (dende*)	Lagenaria siceraria, Containe of groundnut butter
Taro	Colacasia antiquorum, Yam
Mowa	Decumbent perennial weed; Amaranthus thunbergii& graecizans (leaves cooked as spinach)
	
**Meat**	
Caterpillar	Edible caterpillar (dark, with white fur, found on musasa trees. Eaten fried or sun-dried
Matemba, Kapenta Mouse	Small fish caught mostly from lake Kariba Wild mice (not rats or house mice), these are trapped in the wild or fields. Eaten cooked with soup or dried.
	
**Milk and dairy product**	
Lacto	Commercially produced fermented milk and taste sour

* Name of food in Shona

In Table 3 we have presented the reported consumption of selected foods from the FFQ. Almost all the participants reported eating sadza at least 5 times a week, but there was more variation in reported intakes of other foods in that group, for instance porridge (Table 5). About half the participants ate matemba and caterpillar more than once a month, and 44% reported they ate mice at least monthly. The main source of dairy was lacto. Most people ate mango in season and banana the year round. People either never drank coffee or consumed it very frequently (Table 5).

**Table 5:** Reported consumption of selected foods in the past year in Zimbabwe from FFQ

**Foods**	**Frequency of consumption**				
	**Never, <1/mo**	**1–3 / mo**	**1/wk**	**2–4 / wk**	**≥ 5 / wk**
**Bread, cereal and starches**					
Sadza (maize)	0	0	0	4 (2%)	97 (98%)
White bread	40 (40.8%)	16 (16.3%)	4 (4.1%)	7 (7.1%)	31 (31.6%)
**Starch roots and tubers**					
Sweet potato	3 (3%)	9 (9.1%)	9 (9.1%)	32 (32.3%)	44 (44.5%)
**Meat and eggs**					
Matemba	46 (46.5%)	24 (24.2%)	16 (16.2%)	11 (11.1%)	2 (2%)
Caterpillar	52 (52.5%)	28 (28.3%)	5 (5.1%)	12 (12.1%)	2 (2%)
Mice	66 (66.7%)	12 (12.1%)	11 (11.1%)	3 (3%)	7 (7.1%)
**Dairy products**					
Lacto	36 (36.4%)	38 (38.4%)	9 (9.1%)	15 (15.2%)	1 (1%)
Whole milk	79 (79.8%)	9 (9.1%)	2 (2%)	6 (6.1%)	3 (3%)
**Fruits in season**					
Mango	2 (2%)	3 (3%)	12 (12.1%)	19 (19.2%)	63 (63.8%)
Guava	9 (9.1%)	9 (9.1%)	10 (10.1%)	24 (24.2%)	47 (47.5%)
**Fruits out of season**					
Banana	33 (33.3%)	25 (25.3%)	7 (7.1%)	10 (10%)	24 (24.2%)
Paw Paw	77 (77.8%)	16 (16.2%)	3 (3%)	1 (1%)	2 (2%)

Type of oil most commonly used for cooking (N,%) Vegetable oil 98 (99%)

We obtained very similar estimates by the USDA and Zimbabwean food composition tables for energy intake from 30 selected foods (1492 kcal versus 1401 kcal, ICC = 0.99), and carbohydrate (300 g versus 289 g, ICC = 0.99) (Table 6). There were differences in estimates obtained by the USDA and Zimbabwean food composition tables respectively for the micronutrients especially vitamin A (4362 versus 1240 retinol equivalents based on μg, ICC = 0.53), and total folate (99 versus 309 μg/d, ICC = 0.68). A copy of the Zimbabwean food composition table is in Appendix 2.

**Table 6:** Comparison of nutrient intake of 100 participants by USDA and Zimbabwe food composition table

**Nutrients**	USDA Mean (SD)	Zim FCT* Mean (SD)	Intraclass correlation
Energy (kcal)	1492 (661)	1401 (597)	0.99
Protein (g)	33 (15)	46 (25)	0.94
Fat (g)	20 (12)	26 (15)	0.97
Carbohydrate (g)	300 (131)	289 (133)	0.99
Calcium (mg)	443 (222)	360 (226)	0.98
Phosphorus (mg)	560 (250)	987 (367)	0.94
Potassium (mg)	2746 (1830)	3955 (2177)	0.98
Sodium (mg)	1160 (401)	1561 (644)	0.78
Vitamin A, (RE)**	4362 (2978)	1240 (789)	0.53
Vitamin C (mg)	567 (462)	506 (523)	0.99
Total folate (μ-g)	99 (66)	309 (183)	0.68

* FCT = Food composition table

** RE = Retinol equivalents

## Discussion

In this paper we have described the development of an FFQ and a food composition database for the Black Zimbabwean population. We have also provided a copy of the FFQ and food composition table for the benefit of other researchers in the field.

Our sample included a wide cross-section of Zimbabwean society – men, women, rich and poor, urban and rural. We did this to get a list of nutrient rich foods that were eaten by a wide cross-section of people in the community that would help in categorizing people by nutrient intake. There was a wide spread of intakes of porridge and white bread and are examples of discriminating foods (Table 4).

We tried to phrase the questions in the FFQ so that the respondent could picture the food we were asking about in his or her mind. For example the portion size of meat in Zimbabwe is considerably smaller than that in western countries. Matemba (fish) is eaten in very small quantities almost like a condiment. We took these factors into account when designing the questions and analyzing nutrient content. The questions were short, used average portion sizes, and categories of intake so that the interviewer simply checked a category to estimate frequency of intake, and did not have to estimate portion size. As a result its administration (by a trained interviewer) took on average 10 minutes. The instrument has face validity. For example, sadza is the staple food in Zimbabwe and almost all the participants reported that they ate it at least 5 times a week. We are conducting a formal validation of this instrument by comparing nutrient and food intakes assessed from the FFQ to multiple 24-hour recalls.

By using the USDA food composition database as our major source of information we ensured that nutrient estimates were available for most foods, and the assays to estimate nutrients were current [3]. By using the Zimbabwean food composition database as a guide we were able to select the food item from the USDA food composition database that was closest in nutrient content to the corresponding Zimbabwean food. The choice was probably reasonable as the nutrient estimates from the two methods were very similar for most nutrients. The discrepancies, particularly for vitamin A and folate, were probably because certain foods were not analyzed for these nutrients and hence there were missing values in the Zimbabwean table, or old methods of nutrient analysis were used. The methods to estimate vitamin A and folate from foods have changed in the USDA nutrient database [3], but those for macronutrients have not.

Instead of the backward selection procedure to develop a food list, we collected a 24-hour dietary data, supplemented with input from local nutritionists. Such an approach has also been used for the development of FFQs [2]. Moreover the variation of food intake in low income countries is low [2] so it is unlikely that we missed out any important food in the FFQ. The participants in this study were persons over 35 years of age. The FFQ could likely be used among younger adults as well because it probably captures most of the foods eaten by this group as well but this caveat should be borne in mind when extending its use. Another limitation was that we did not have nutrient contents of certain foods, for example, caterpillar. We overcame this by using the closest category (sushi caterpillar) that was in the ESHA food composition table to that food. These assumptions would inevitably be the source of some errors in nutrient estimation.

The discrepancies in nutrient estimates obtained from the same FFQ data but different food composition tables underscores the need to have standard methods, not only to develop the FFQ, but also food composition tables. While there is much literature on methods for FFQ development there is much less information on food composition database development. We have described a standardized process of FFQ and food composition database development for a Black Zimbabwean population.

## List of abbreviations

FFQ Food Frequency Questionnaire

24 hr DR 24 hour Dietary Recall

USDA United State Department of Agriculture

SPSS Statistical Packages for Social Sciences

ESHA The Food Processor and Genesis

SD Standard deviation

## Authors' contributions

ATM Participated in design of study, performed statistical analysis, drafted the manuscript

MD Participated in design of study, coordinated and performed statistical analysis, helped to draft the manuscript

JC Coordinated study in Zimbabwe, helped to draft the manuscript

GT Facilitated data collection in Zimbabwe

SY Participated in securing funding, helped to draft the manuscript

All authors read and approved the final manuscript.

**Figure 1:**
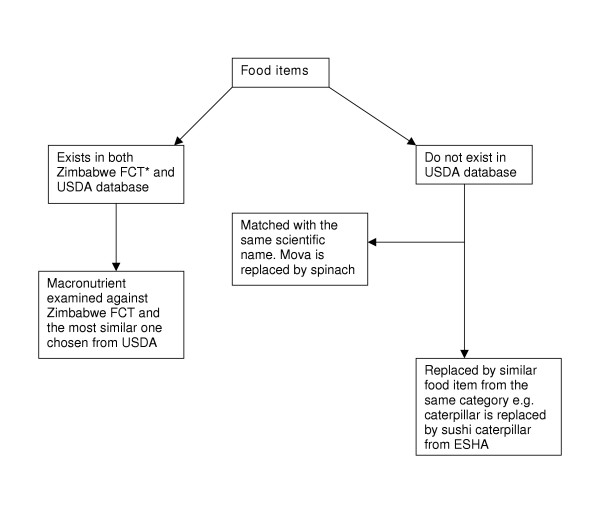
The process of choosing Food items from USDA nutrient database. (FCT* : Food Composition Table) ESHA: Computer program to estimate nutrient content of foods

## Supplementary Material

Additional File 1 pdf file containing the FFQ for Zimbabwe Click here for file

Additional File 2 Excel file containing the food composition database for Zimbabwe Click here for file

## References

[B1] Popkin BM (1999). Urbanization, lifestyle changes and the nutrition transition. World Development.

[B2] Willett W, Willett W (1998). Food Frequency Methods. Nutritional epidemiology.

[B3] (2004). USDA National Nutrient Database for Standard Reference Release 17. http://www.nal.usda.gov/fnic/foodcomp.

[B4] Chitsiku IC (2000). Nutritive Value of Foods in Zimbabwe.

[B5] Rimm EB, Giovannucci EL, Stampfer MJ, Colditz GA, Litin LB, Willett WC (1992). Reproducibility and validity of an expanded self-administered semiquantitative food frequency questionnaire among male health professionals. Am J Epidemiol.

[B6] (2004). ESHA: The food Processor.

[B7] Dehghan M, Al Hamad N, Yusufali A, Nusrath F, Yusuf S, Merchant AT (2005). Development of a semi-quantitative food frequency questionnaire for use in United Arab Emirates and Kuwait based on local foods. Nutr J.

