# Supplementary material for: Nutrient estimation from an FFQ developed for a black Zimbabwean population
Source: Nutr J. 2005 Dec 13;4:37. doi: 10.1186/1475-2891-4-37 (PMC1325035; doi:10.1186/1475-2891-4-37)
Supplement: Additional File 1 — pdf file containing the FFQ for Zimbabwe [file 1475-2891-4-37-S1.pdf]

PURE/Zimbabwe

We are very grateful to you for your participation in this study. All information given by you will be held in strict confidence, and will be used for the purpose of this study only after removing any personal identifying information.

Adult Semi-Quantitative Food Frequency Questionnaire

INSTRUCTIONS

Please answer EACH question by marking an X in ONE BOX on each line: (unless otherwise instructed)

[X]

OR

By writing number(s) in the spaces provided:

[1] [8]

OR

By specifying the answer on the line(s) provided

☐☐	☐☐☐	☐☐☐	☐☐	**Subject Initials** ☐☐☐
Centre #	*Community#*	*Household #*	*Subject #*	

Today's date: 2 0 ☐☐ ☐☐ ☐☐
year *month* *day*

During the past year, on average, how often have you consumed the following dairy foods? (please check the appropriate box)

**Dairy Foods**	**Avg Serving**	**Never, less than once/month**	**1-3/mo**	**1/wk**	**2-4/wk**	**5-6/wk**	**1/day**	**2-3/day**	**4-5/day**	**>6/day**
**1.** Whole milk	1 glass	☐	☐	☐	☐	☐	☐	☐	☐	☐
**2.** Skimmed milk	1 glass	☐	☐	☐	☐	☐	☐	☐	☐	☐
**3.** Lacto	1 glass	☐	☐	☐	☐	☐	☐	☐	☐	☐
**4.** Cascade	1 glass	☐	☐	☐	☐	☐	☐	☐	☐	☐
**5.** Cheese	30g	☐	☐	☐	☐	☐	☐	☐	☐	☐
**6.** Ice cream	1 cup	☐	☐	☐	☐	☐	☐	☐	☐	☐
**7.** Yogurt	1 cup	☐	☐	☐	☐	☐	☐	☐	☐	☐
**8.** Powdered milk for tea	1 tsp	☐	☐	☐	☐	☐	☐	☐	☐	☐
**9.** Margerine added to food or bread	1 pat	☐	☐	☐	☐	☐	☐	☐	☐	☐
**10.** Butter added to food or bread (exclude use in cooking)	1pat	☐	☐	☐	☐	☐	☐	☐	☐	☐

During the past year, on average, how often have you consumed the following fruits? (please check the appropriate box)

**Fruits**										
**11.** Bananas	1 medium	☐	☐	☐	☐	☐	☐	☐	☐	☐
**12.** Paw Paw	1 medium	☐	☐	☐	☐	☐	☐	☐	☐	☐

Centre #	*Community#*	*Household #*	*Subject #*	**Subject Initials**

When in season, how often do you eat the following fruits over a period of one month?
(Please check the appropriate box)

**Fruits**	**Avg Serving**	**Never, less than once/month**	**1-3/mo**	**1/wk**	**2-4/wk**	**5-6/wk**	**1/day**	**2-3/day**	**4-5/day**	**>6/day**
**13.** Baobab	1 medium	☐	☐	☐	☐	☐	☐	☐	☐	☐
**14.** Guava	1 medium	☐	☐	☐	☐	☐	☐	☐	☐	☐
**15.** Mango	1 medium	☐	☐	☐	☐	☐	☐	☐	☐	☐
**16.** Naartjie	1 medium	☐	☐	☐	☐	☐	☐	☐	☐	☐
**17.** Apple	1 medium	☐	☐	☐	☐	☐	☐	☐	☐	☐
**18.** Avocado	1/2 fruit	☐	☐	☐	☐	☐	☐	☐	☐	☐
**19.** Orange	1 medium	☐	☐	☐	☐	☐	☐	☐	☐	☐
**20.** Lemon	1 fruit	☐	☐	☐	☐	☐	☐	☐	☐	☐
**21.** Grape	1/2 cup	☐	☐	☐	☐	☐	☐	☐	☐	☐

When in season, how often do you eat the following fruits over a period of one month?
(Please check the appropriate box)

**22.** Peaches	1 fresh	☐	☐	☐	☐	☐	☐	☐	☐	☐
**23.** Sugar Cane	1 piece	☐	☐	☐	☐	☐	☐	☐	☐	☐
**24.** Chakata	1/2 cup	☐	☐	☐	☐	☐	☐	☐	☐	☐
**25.** Mazhanje	1/2 cup	☐	☐	☐	☐	☐	☐	☐	☐	☐
**26.** Pineapple	1/2 cup	☐	☐	☐	☐	☐	☐	☐	☐	☐
**27.** Matohwe	1/2 cup	☐	☐	☐	☐	☐	☐	☐	☐	☐

Centre #	Community#	Household #	Subject #

Subject Initials

During the past year, on average, how often have you consumed the following vegetables? (please check the appropriate box)

	Avg Serving	Never, less than once/month	1-3/mo	1/wk	2-4/wk	5-6/wk	1/day	2-3/day	4-5/day	>6/day
**Vegetables**										
**28.** Carrot (cooked)	1/2 cup	☐	☐	☐	☐	☐	☐	☐	☐	☐
**29.** Carrot (fresh)	1/2 cup	☐	☐	☐	☐	☐	☐	☐	☐	☐
**30.** Beans or lentils, baked or dried	1/2 cup	☐	☐	☐	☐	☐	☐	☐	☐	☐
**31.** Cucumber	1/2 cup	☐	☐	☐	☐	☐	☐	☐	☐	☐
**32.** Mushrooms, fresh, cooked or canned	1/2 cup	☐	☐	☐	☐	☐	☐	☐	☐	☐
**33.** Nyevhe	1/2 cup	☐	☐	☐	☐	☐	☐	☐	☐	☐
**34.** Muboora	1/2 cup	☐	☐	☐	☐	☐	☐	☐	☐	☐
**35.** Spinach (cooked)	1/2 cup	☐	☐	☐	☐	☐	☐	☐	☐	☐
**36.** Mowa	1/2 cup	☐	☐	☐	☐	☐	☐	☐	☐	☐
**37.** Pepper (cooked)	1/2 cup	☐	☐	☐	☐	☐	☐	☐	☐	☐
**38.** Taro	1/2 cup	☐	☐	☐	☐	☐	☐	☐	☐	☐
**39.** Mufushma (dried)	1/2 cup	☐	☐	☐	☐	☐	☐	☐	☐	☐
**40.** Mufushma (cooked)	1/2 cup	☐	☐	☐	☐	☐	☐	☐	☐	☐

When in season, how often do you eat the following vegetables over a period of one month?

(Please check the appropriate box)

**41.** Okra	1/2 cup	☐	☐	☐	☐	☐	☐	☐	☐	☐

Centre #	Community#	Household #	Subject #	Subject Initials
☐☐	☐☐☐	☐☐☐	☐☐	☐☐☐

	Avg Serving	Never, less than once/month	1-3/mo	1/wk	2-4/wk	5-6/wk	1/day	2-3/day	4-5/day	>6/day
**42.** Lima beans fresh,frozen or canned)	1/2 cup	☐	☐	☐	☐	☐	☐	☐	☐	☐
**43.** Cabbage (cooked)	1/2 cup	☐	☐	☐	☐	☐	☐	☐	☐	☐
**44.** Cabbage (fresh)	1/2 cup	☐	☐	☐	☐	☐	☐	☐	☐	☐
**45.** Celery	4 inch stick	☐	☐	☐	☐	☐	☐	☐	☐	☐
**46.** Mushamba	1/2 cup	☐	☐	☐	☐	☐	☐	☐	☐	☐
**47.** Gourd	1/2 cup	☐	☐	☐	☐	☐	☐	☐	☐	☐
**48.** Peas (cooked)	1/2 cup	☐	☐	☐	☐	☐	☐	☐	☐	☐
**49.** Pumpkin (cooked)	1/2 cup	☐	☐	☐	☐	☐	☐	☐	☐	☐
**50.** Turnip	1/2 cup	☐	☐	☐	☐	☐	☐	☐	☐	☐
**51.** Green vegetables	1/2 cup	☐	☐	☐	☐	☐	☐	☐	☐	☐

During the past year, on average, how often have you consumed the following meat, eggs, etc.? (please check the appropriate box)

<u>Meat, eggs etc.</u>

	Avg Serving	Never, less than once/month	1-3/mo	1/wk	2-4/wk	5-6/wk	1/day	2-3/day	4-5/day	>6/day
**52.** Eggs	1	☐	☐	☐	☐	☐	☐	☐	☐	☐
**53.** Chicken or turkey, with skin	90-120 g	☐	☐	☐	☐	☐	☐	☐	☐	☐
**54.** Birds	90-120 g	☐	☐	☐	☐	☐	☐	☐	☐	☐
**55.** Beef, pork, goat or lamb as a sandwich or mixed dish (eg. stew, casserole, lasagna etc.)	90-120 g	☐	☐	☐	☐	☐	☐	☐	☐	☐

Centre # | Community# | Household # | Subject # | Subject Initials

**Meat, eggs etc con't.**	**Avg Serving**	**Never, less than once/month**	**1-3/mo**	**1/wk**	**2-4/wk**	**5-6/wk**	**1/day**	**2-3/day**	**4-5/day**	**>6/day**
**56.** Beef, pork, goat lamb as a main dish (eg. steak roast, ham etc.)	90-120 g	☐	☐	☐	☐	☐	☐	☐	☐	☐
**57.** Matemba	90-120 g	☐	☐	☐	☐	☐	☐	☐	☐	☐
**58.** Canned tuna fish	90-120 g	☐	☐	☐	☐	☐	☐	☐	☐	☐
**59.** Rabbit	90-120 g	☐	☐	☐	☐	☐	☐	☐	☐	☐
**60.** Mice	100 g	☐	☐	☐	☐	☐	☐	☐	☐	☐
**61.** Caterpillar	90-120 g	☐	☐	☐	☐	☐	☐	☐	☐	☐
**62.** Locust	90-120 g	☐	☐	☐	☐	☐	☐	☐	☐	☐
**63.** Termites	90-120 g	☐	☐	☐	☐	☐	☐	☐	☐	☐
**64.** Fish	90-120 g	☐	☐	☐	☐	☐	☐	☐	☐	☐
**65.** Snake	90-120 g	☐	☐	☐	☐	☐	☐	☐	☐	☐

During the past year, on average, how often have you consumed the following breads, cereals and starches? (please check the appropriate box)

**Breads, cereals, starches**										
**66.** Sadza from maize	1 plate	☐	☐	☐	☐	☐	☐	☐	☐	☐
**67.** Sadza from millet	1 plate	☐	☐	☐	☐	☐	☐	☐	☐	☐
**68.** Sadza from sorghum	1 plate	☐	☐	☐	☐	☐	☐	☐	☐	☐
**69.** Samp	40g	☐	☐	☐	☐	☐	☐	☐	☐	☐

☐☐	☐☐☐	☐☐☐	☐☐	**Subject Initials** ☐☐☐
Centre #	*Community#*	*Household #*	*Subject #*	

**Bread, cereals con't.**	**Avg Serving**	**Never, less than once/month**	**1-3/mo**	**1/wk**	**2-4/wk**	**5-6/wk**	**1/day**	**2-3/day**	**4-5/day**	**>6/day**
**70.** White bread	1 slice	☐	☐	☐	☐	☐	☐	☐	☐	☐
**71.** Dark bread	1 slice	☐	☐	☐	☐	☐	☐	☐	☐	☐
**72.** Mealie	1 plate	☐	☐	☐	☐	☐	☐	☐	☐	☐
**73.** Biscuits	1	☐	☐	☐	☐	☐	☐	☐	☐	☐
**74.** Porridge (maize, corn)	1 cup	☐	☐	☐	☐	☐	☐	☐	☐	☐
**75.** White rice (cooked)	1 cup	☐	☐	☐	☐	☐	☐	☐	☐	☐
**76.** Macaroni (cooked)	1 cup	☐	☐	☐	☐	☐	☐	☐	☐	☐
**77.** Samosa	1 piece	☐	☐	☐	☐	☐	☐	☐	☐	☐
**78..** Nophi	1 piece	☐	☐	☐	☐	☐	☐	☐	☐	☐

During the past year, on average, how often have you consumed the following starchy roots and tubers? (please check the appropriate box)

**Starchy roots and tubers**										
**79.** Potatoes	1 cup	☐	☐	☐	☐	☐	☐	☐	☐	☐
**80.** Sweet Potatoes	1 cup	☐	☐	☐	☐	☐	☐	☐	☐	☐
**81.** Cassava	1 cup	☐	☐	☐	☐	☐	☐	☐	☐	☐

During the past year, on average, how often have you consumed the following beverages? (please check the appropriate box)

**Beverages**										
**82.** Orange juice	1 cup	☐	☐	☐	☐	☐	☐	☐	☐	☐
**83.** Coffee	1 cup	☐	☐	☐	☐	☐	☐	☐	☐	☐

Centre #	Community#	Household #	Subject #	Subject Initials

**Beverages con't**	**Avg Serving**	**Never, less than once/month**	**1-3/mo**	**1/wk**	**2-4/wk**	**5-6/wk**	**1/day**	**2-3/day**	**4-5/day**	**>6/day**
**84.** Tea not herbal	1 cup	☐	☐	☐	☐	☐	☐	☐	☐	☐
**85.** Maheu	1 cup	☐	☐	☐	☐	☐	☐	☐	☐	☐
**86.** Traditional beer	1 pint	☐	☐	☐	☐	☐	☐	☐	☐	☐
**87.** Gingerale, Fanta Freezit (regular)	1 bottle	☐	☐	☐	☐	☐	☐	☐	☐	☐
**88.** Gingerale, Fanta, Freezit (light)	1 bottle	☐	☐	☐	☐	☐	☐	☐	☐	☐

During the past year, on average, how often have you consumed the following sweet baked goods? (please check the appropriate box)

**Sweet, Baked goods**										
**89.** Candy	40 g	☐	☐	☐	☐	☐	☐	☐	☐	☐
**90.** Cake (home made)	1 slice	☐	☐	☐	☐	☐	☐	☐	☐	☐
**91.** Peanut butter	1 tbs.	☐	☐	☐	☐	☐	☐	☐	☐	☐
**92.** Seeds	1 tbs.	☐	☐	☐	☐	☐	☐	☐	☐	☐
**93.** Nuts	1 tbs.	☐	☐	☐	☐	☐	☐	☐	☐	☐

Vitamins

94. Do you regularly take vitamin pills? ☐ No ☐ Yes

95. Do you regularly take mineral pills? ☐ No ☐ Yes

96. If yes for how many years and how often have you taken?

	**< 1 year**		**1-3 years**		**4-6 years**		**> 6 years**	
	**mostly**	**occasionaly**	**mostly**	**occasionaly**	**mostly**	**occasionaly**	**mostly**	**occasionaly**
**97.** . Vitamin A	☐	☐	☐	☐	☐	☐	☐	☐
**98.** Vitamin D	☐	☐	☐	☐	☐	☐	☐	☐

Centre #	Community#	Household #	Subject #

Subject Initials

	< 1 year		1-3 years		4-6 years		> 6 years	
	**mostly**	**occasionaly**	**mostly**	**occasionaly**	**mostly**	**occasionaly**	**mostly**	**occasionaly**
**99.** Vitamin E	☐	☐	☐	☐	☐	☐	☐	☐
**100.** Vitamin B complex	☐	☐	☐	☐	☐	☐	☐	☐
**101.** Vitamin C	☐	☐	☐	☐	☐	☐	☐	☐
**102.** Calcium	☐	☐	☐	☐	☐	☐	☐	☐
**103.** Iron	☐	☐	☐	☐	☐	☐	☐	☐
**104.** Zinc	☐	☐	☐	☐	☐	☐	☐	☐
**105.** Multiviamin /Mineral supplement	☐	☐	☐	☐	☐	☐	☐	☐

106. What type of fat/oil do you use most often for cooking?

☐ Vegetable oil ☐ Canola /rape seed oil ☐ Ghee ☐ Lard

107. Name of Interviewer: ______________________ ________
(please print) *Last Name* *First Initial*

Interviewer Code: ☐☐☐
